# Supplementary material for: A Scoping Review of Preterm Births in Sub-Saharan Africa: Burden, Risk Factors and Outcomes
Source: Int J Environ Res Public Health. 2022 Aug 24;19(17):10537. doi: 10.3390/ijerph191710537 (PMC9518061; doi:10.3390/ijerph191710537)
Supplement: Supplementary file 1 [file ijerph-19-10537-s001.zip › ijerph-1837961-supplementary.pdf]

Table S1. Search String used in the PubMed Database.

(Preterm Birth OR Birth, Preterm OR Births, Preterm OR infants, Premature OR Premature Infant OR Preterm Infant OR Infant, Preterm OR Infants, Preterm OR Preterm Infant OR Premature Infants OR Neonatal Prematurity OR Prematurity, Neonatal) AND ("Africa south of the Sahara" Angola/ OR Benin/ OR Botswana/ OR Burkina Faso/ OR Burundi/ OR Cameroon/ OR cape Verde/ OR central Africa/ OR central African republic/ OR Chad/ OR Comoros/ OR Congo/ OR cote d'ivoire/ OR democratic republic Congo/ OR Djibouti/ OR equatorial guinea/ OR Eritrea/ OR Ethiopia/ OR Gabon/ OR Gambia/ OR Ghana/ OR guinea/ OR Guinea-Bissau/ OR Kenya/ OR Lesotho/ OR Liberia/ OR Madagascar/ OR Malawi/ OR Mali/ OR Mayotte/ OR Mozambique/ OR Namibia/ OR Niger/ OR Nigeria/ OR Rwanda/ OR Sahel/ OR Senegal/ OR Sierra Leone/ OR Somalia/ OR south Africa/ OR south Sudan/ OR Sudan/ OR Swaziland/ OR Tanzania/ OR Togo/ OR Uganda/ OR Zambia/ or Zimbabwe/)

Table S2. Characteristics of included studies.

First author, year	Country	Study design	Population	Place of delivery	Sample Size	Measures of gestational age	Prevalence of PTB (%)	Outcomes
Abadiga et al., 2021	Ethiopia	Case control	General population of pregnant women	Public hospitals	516	LMP, USS	NR	NR
Abaraya et al., 2018	Ethiopia	Cohort	General population of pregnant women	Hospital	656	NR	11.8	NR
Abdo et al.,, 2020	Ethiopia	Cross-sectional	General population of pregnant women	Hospital	304	LMP, USS	15.5	NR
Abdul-mumin et al., 2020	Ghana	Cohort	Preterm babies admitted	Hospital	192	NR	NR	-Participants were hospitalized preterm babies.<br>-39.3% of these babies died.<br>-87.0% of extremely preterm babies died during hospitalization
Abebe et al., 2020	Ethiopia	Cohort	Teenage pregnant women	Hospital	309	NR	12.9	NR
Abrams et al., 2004	Malawi	Cross-sectional	General population of pregnant women	Hospital	572	Ballard score	8.2	NR
Adam et al., 2009	Sudan	Cross-sectional	General population of pregnant women	Hospital	644	LMP, USS	4.5	NR
Adane et al., 2014	Ethiopia	Cross-sectional	General population of pregnant women	Hospital	490	LMP, USS	14.3	
Adeniran et al, 2014	Nigeria	Case control	HIV positive pregnant women	Hospital	268	NR	7.8	
Adesina et al., 2015	Nigeria	Cross-sectional	HIV positive pregnant women	Hospital	307	NR	35.8	
Adu-Bonsaffoh et al., 2019	Ghana	Cohort	General population of pregnant women	Hospital	7801	LMP	18.9	-Premature babies were more likely to be stillbirths, have poor APGAR score, and be admitted to NICU
Agbeno et al., 2021	Ghana	Cohort	Hospitalized preterm babies	Hospital	27,320	NR	4.7	-Participants were hospitalized preterm babies.<br>-27.6% of preterm infants died.

								-Survival depended on birth weight (p <0.001); gestational age (p <0.001); mode and place of delivery (p <0·001 for both); APGAR scores at 1st and 5th minutes (p <0.001), and length of stay at the SCBU (p <0.001)·
Agbla et al., 2006	Benin	Case control	General population of pregnant women	Hospital	203	LMP, USS	NR	NR
Akintayo et al., 2015	Nigeria	Case control	General population of pregnant women	Hospital	4347	LMP	6.0	-Premature babies were more likely to be admitted to special baby care units, have poor APGAR scores, and had higher perinatal mortality
Akintije et al., 2020	Rwanda	Cross-sectional	General population of pregnant women	Hospital	4960	LMP	9.9 overall, 21.6 among those with one ANC visit, 22.3 among those with two ANC visits, 17.5 among those with three ANC visits, and 5.6 among those with four ANC visits	NR
Alhaj et al., 2010	Sudan	Case control	General population of pregnant women	Hospital	3240	LMP, USS	3.8	NR
Aniji et al., 2012	South Africa	Cohort	HIV positive pregnant women	Hospital	245	USS	23.2	NR
Anthony et al., 2020	Malawi	Cross-sectional	General population of pregnant women	Health facilities and community	14,792	LMP, USS, SFH	19.3	NR
Aregawi et al., 2019	Ethiopia	Cross-sectional	General population of pregnant women	Hospital	472	LMP	13.3	NR
Asefa et al., 2020	Ethiopia	Cross-sectional	General population of pregnant women	Hospital	752	NR	6.9 overall 11.2 among advanced-aged mothers	NR

							2.6 among younger-aged mothers	
Aseidu et al., 2019	Ghana	Case control	General population of pregnant women	Hospital	390	NR	NR	NR
Bako et al., 2009	Nigeria	Cohort	General population of pregnant women	Hospital	400	NR	7.0	NR
Bako et al, 2017	Nigeria	Case control	Preterm babies admitted	Hospital	379	LMP, USS	NR	-Participants were hospitalized preterm babies.<br>-19.6% of these babies died
Balkus et al., 2021	Malawi<br>South Africa<br>Uganda<br>Zimbabwe	Cross-sectional	General population of pregnant women	Hospitals	10,138	LMP, USS, SFH	13.0 overall<br>10.7 in Malawi<br>20.7 in South Africa<br>10.4 in Uganda,<br>11.7 in Zimbabwe	NR
Bater et al., 2020	Uganda	Cohort	General population of pregnant women	NR	3841	LMP	19.4	NR
Bello et al.,2019	Nigeria	Cohort	Hospitalized preterm babies	Hospital	3435	LMP, USS, Ballard Score	32.9	-Participants were hospitalized preterm babies.<br>-14.7% of these babies died
Berhane et al., 2019	Ethiopia	Cohort	General population of pregnant women	Hospital	1486	LMP, USS, Ballard Score	10.2	
Berhanie et al., 2019	Ethiopia	Case control	General population of pregnant women	Hospital	954	NR	NR	NR
Berhe et al., 2019	Ethiopia	Cohort	Mothers with PIH and comparison peers	Hospital	782	NR	40.8 among mothers with PIH,<br>5.6 among normotensive mothers	NR

Berhe, 2019, Ethiopia	Ethiopia	Cross-sectional	General population of pregnant women	Hospitals	413	NR	12.8	NR
Bisio et al., 2015	Republic of Congo	Cohort	HIV Positive pregnant women	Health centres	188	LMP, USS	10.7	NR
Brabin et al., 2019	Burkina Faso	RCT	Nulliparous women	Health centres	286	USS	27.5 among cases<br>13.9 among controls	NR
Brhane et al., 2019	Ethiopia	Cohort	General population of pregnant women	Health facilities	460	LMP	10.4 overall<br>25.9 among mothers with short IPI<br>2.9 among mothers with recommended IPI	NR
Brink et al., 2020		Cohort	General population of pregnant women	Clinics	5806	LMP	13.3	NR
Brits et al., 2015	South Africa	Case control	General population of pregnant women	Hospital	198	LMP	NR	NR
Brittain et al., 2015	South Africa	Cohort	General population of pregnant women	Clinics	726	USS, SFH	15.0	NR
Butali et al., 2016	Nigeria	Cross-sectional	General population of pregnant women	Hospital	4691	LMP, USS	16.8	NR
Chen et al., 2012	Botswana	Cohort	HIV positive pregnant women	Hospitals	9504	LMP	19.6	NR
Chetty et al., 2018	South Africa	Cohort	HIV Positive pregnant women	Clinics	2549	LMP, USS, SFH	10.4	NR
Chiabi et al., 2013	Cameroon	Cross-sectional	Preterm babies admitted and comparison peers	Hospital	1066	LMP	26.5	-Participants were hospitalized preterm babies and comparison peers.<br>-36.6% of preterm babies died, in which 69% occurred in the early neonatal period.<br>-The main causes of death were neonatal infections (27.6%), neonatal asphyxia (11·9%) and congenital malformations (10·3%) .

Conroy et al., 2017	Uganda	RCT	HIV Positive pregnant women	Clinics	326	LMP, USS	16.9	NR
Dadabhai et al., 2019	Malawi	Cohort	General population of pregnant women	Health facilities	1299	LMP, Ballard score	10.6 among HIV infected women<br>9.5 among HIV uninfected women	NR
Dadi et al., 2020	Ethiopia	Cohort	General population of pregnant women	NR	916	LMP, USS	16.4	NR
D'Cruz et al., 2012	Tanzania	Case control	General population of pregnant women	Hospital	188	LMP	26.1	NR
De Beaudrap et al., 2013	Uganda	Cohort	General population of pregnant women	Hospital	1218	USS, SFH	7.0	NR
Debelo et al., 2020	Ethiopia	Cohort	Pregnant mothers with advanced age	Hospital	907	NR	19.8	NR
Deressa et al., 2018	Ethiopia	Cross-sectional	Babies admitted	Hospital	23,115	NR	16.5	NR
Dettinger et al., 2019	Kenya	Case control	Mothers with prenatal PrEP exposure and comparison peers	Health clinics	1530	LMP	3.4 among mothers with PrEP exposure,<br>6.9 among controls	NR
Egbe et al., 2015	Cameroon	Cross-sectional	Adolescent pregnant women	Hospital	148	LMP	13.0	NR
Ejigu et al., 2019	Ethiopia	Cohort	HIV Positive pregnant women on ART	Hospital	1663	LMP, USS, SFH	16.6	NR
Elphinstone et al., 2019	Malawi	Cohort	HIV negative women	Hospital	1506	USS	20.2	NR
Ekouevi et al., 2011	Côte d'Ivoire	Cohort	HIV Positive pregnant women	NR	344	LMP, USS	11.0	NR
Etuk et al., 2005	Nigeria	Cross-sectional	General population of pregnant women	Hospital	2640	NR	9.0	NR
Ezechi et al., 2012	Nigeria	Cohort	HIV positive pregnant women	Hospital	1626	LMP	11.0	NR

Ezegwui et al., 2012	Nigeria	Case control	General population of pregnant women	Hospital	179	LMP	17.0	NR
Ezenwa et al., 2021	Nigeria	Cross-sectional	General population of pregnant women	Both home and health facility	628	Ballard score	29.3	NR
Fajolu et al., 2019	Nigeria	Cohort	General population of pregnant women	Hospital	11,607	LMP, USS, Ballard Score	14.5	-Target participants were hospitalized extremely preterm babies.<br>-75.7% of these babies died
Fawcus et al., 1992	Zimbabwe	Case control	General population of pregnant women	Hospital	380	NR	25.0	NR
Fawzi et al., 1998	Tanzania	RCT	HIV positive pregnant women	Hospital	888	LMP	23.0	NR
Fawzi et al., 2007	Tanzania	RCT	HIV negative pregnant women	Hospital	8468	LMP	17.0	NR
Fennell et al., 2021	Botswana	Cohort	HIV positive pregnant women	Hospital	8867	LMP, USS	21.6	NR
Feresu et al., 2010	Zimbabwe	Cohort	General population of pregnant women	Hospital	17,174	LMP	22.0	NR
Feresu et al., 2004	Zimbabwe	Case control	General population of pregnant women	Hospital	3103	Ballard score	16.0	NR
Fouelifack et al., 2014		Cross-sectional	General population of pregnant women	Hospital	4032	NR	25.0	NR
Garcia-Basteiro et al., 2017	Mozambique	Cohort	General population of pregnant women	Hospital	5574	Ballard score, Dubowitz	6.2	-Neonatal and infant mortality rate for preterm babies was 980.2/ 1000 and 136.1/1000 children years at risk, respectively<br>-Preterm babies had 13 times higher risk of mortality compared to 5 times in term babies<br>-Risk of hospitalization higher in preterm babies
Gejo et al., 2021	Ethiopia	Case control	General population of pregnant women	Hospital	213	LMP, USS	NR	NR

Gesase et al., 2018	Tanzania	Cross-sectional	General population of pregnant women	Hospital	1117	NR	14.0	NR
Gibango et al., 2018	South Africa	Cross-sectional	HIV positive women on antiretroviral treatment	Hospital	496	Ballard score	32.0	NR
Gibb et al., 2012	Zimbabwe Uganda	RCT	HIV positive pregnant women	Hospital	226	LMP	10.0	NR
Gladstone et al., 2011	Malawi	Cohort	General population of pregnant women	65.5% in health facility	840	USS	NR	-Greater risk of death among preterm babies during the postnatal period (HR1.8, 1.1-3.0).<br>- Surviving preterm infants were more likely to be underweight (weight-for-age z score; $p<0.001$) or wasted (weight-for-length z score; $p<0.01$).<br>- Preterm infants more often screened positively for disability on the Ten Question Questionnaire ($p=0.002$).<br>- They also had higher rates of developmental delay on the Malawi Developmental Assessment Tool at 18 months ($p=0.009$).<br>-Morbidity (hospital visits and hospitalizations) were similar for preterm and term babies.
Govender et al., 2018	South Africa	Cross-sectional	Adolescent pregnant women	Hospital	314	NR	6.0	NR
Gumede et al., 2017	South Africa	Cross-sectional	General population of pregnant women	Hospital	31,179	NR	17.0	NR
Habib et al., 2008	Tanzania	Cohort	General population of pregnant women	Hospital	14,444	LMP	9.0	NR
Harjunmaa et al., 2015	Malawi	Cross-sectional	General population of pregnant women	Home and health facility	1024	USS	8.0	NR
Hassen et al., 2021	Ethiopia	Case control	General population of pregnant women	Hospital	365	LMP, USS	24.9	NR

Hoque et al., 2014	South Africa	Cohort	General population of pregnant women	Health facility	2325	NR	16.0	NR
Hussain et al., 2011	South Africa	Cross-sectional	General population of pregnant women	Hospital	1622	Ballard score	22.0	NR
Hussein et al., 2009	Tanzania	Cross-sectional	General population of pregnant women	Hospital	1721	LMP, USS	17.0	NR
Hussein et al., 2020	Ghana	Cohort	General population of pregnant women	Hospital	1323	NR	36.4	NR
Iyoke et al., 2015	Nigeria	Cohort	General population of pregnant women	Hospital	784	LMP	17.0	-Survival to discharge rate of preterm babies 38·4% compared to 88.2% for term babies.<br>-Babies born via Vaginal delivery, extreme and very preterm babies had decreased odds of survival.<br>-294 preterm babies were admitted compared to 34 term babies.<br>- The mean duration of admission for singleton preterm babies was 16±5.8 days compared to 5±3.4 days for term babies.
Johnson et al., 2016	Botswana	Cohort	General population of pregnant women	Hospital	14,170	LMP	16.0	NR
Kalanda et al., 2006	Malawi	Cross-sectional	General population of pregnant women	Hospital	1571	Ballard score, Dubowitz	17.3	NR
Kalengo et al., 2020	Tanzania	Cohort	Women with two consecutive singleton births	NR	5946	NR	11.0	NR
Kamfwa et al., 2017	Zambia	Case control	Preterm neonates who died and comparison peers	Hospital	208	NR	NR	-Outcome was early neonatal death and its predictors. LBW was a major predictor of Preterm early neonatal death (aOR132, 39·5-387·7)
Kassa et al., 2019	Ethiopia	Cohort	General population of pregnant women	Hospital	1134	NR	10.0	NR

Kelkay et al., 2018	Ethiopia	Cross-sectional	General population of pregnant women	NR	325	LMP, USS	17.0	NR
Kigozi et al., 2003	Uganda	RCT	Pregnant women with *T· vaginalis*	Hospita	277	LMP, Ballard score	21.0	NR
Kilewo et al., 200	Tanzania Malawi Zambia	Cohort	HIV Positive pregnant women	Hospital	2126	SFH	26.0	NR
Kongnyuy et al., 2008	Cameroon	Cross-sectional	General population of pregnant women	Hospital	1100	NR	15.0	NR
Koss et al., 2014	Uganda	RCT	HIV Positive pregnant women	Hospital	356	LMP, USS	15.0	NR
Kumwenda et al., 2017	Zambia	Cross-sectional	Adolescent pregnant women	Hospital	200	NR	NR	NR
Kuti et al., 2003	Nigeria	Cohort	General population of pregnant women	Hospital	3777	LMP, USS, Ballard score	13.0	-Neonatal mortality rate among term and preterm babies was 3/1000 and 178/1000 children years at risk, respectively.<br>-All extreme preterm babies died.<br>-Major causes of death were RDS (50%), infection (20%), birth asphyxia (15%) and jaundice (10%)
Laelago et al., 2017	Ethiopia	Cross-sectional	General population of pregnant women	Public health facility	183	NR	NR	NR
Letamo et al., 2001	Botswana	Cohort	General population of pregnant women	Health facility	7265	LMP	18.0	NR
Li et al., 2016	Tanzania	Cohort	HIV positive pregnant women	NR	3314	LMP	29.0	NR
Magadi et al., 2006	Kenya	Cross-sectional	Pregnant adolescent girls	Health facility and home delivery	269	LMP	41.7 among urban adolescents 49.4 among rural adolescents	NR
Mahande et al., 2016	Tanzania	Cohort	General population of pregnant women	Hospital	17,030	LMP	10.0	NR

Mahande et al., 2013	Tanzania	Cohort	General population of pregnant women	Hospital	3909	LMP	11.0	NR
Mahande et al., 2013	Tanzania	Cohort	Primiparous women	Hospital	3359	LMP	14.0	NR
Mahande et al., 2016	Tanzania	Cross-sectional	General population of pregnant women	Hospital	3,0797	LMP	10.0	NR
Mahapula et al., 2016	Tanzania	Case control	General population of pregnant women	Hospital	754	NR	NR	NR
Malaba et al, 2018	South Africa	Cohort	General population of pregnant women	Hospital	1787	LMP, USS, SFH	36.0 by LMP, 17.0 by SFH, 11.0 by USS	NR
Malaba et al., 2017	South Africa	Cohort	General population of pregnant women	Hospital	1554	LMP, USS, SFH	21.0	NR
Malaba et al., 2020	South Africa	Cohort	General population of pregnant women	Hospital	1583	LMP, USS, SFH	18.0	NR
Mashau et al., 2020	South Africa	Cohort	General population of pregnant women	NR	198	LMP	9.7	NR
Maymunah et al., 2014	Nigeria	Cohort	General population of pregnant women	Hospital	320	LMP, USS	8.0	NR
Mboya et al., 2021	Tanzania	Cohort	General population of pregnant women	Hospital	44,117	LMP	11.7	NR
Mehari et al., 2020	Ethiopia	Cross-sectional	General population of pregnant women	Hospital	752	NR	7.0	NR
Mekonen et al., 2019	Ethiopia	Cross-sectional	General population of pregnant women	Health institutions	575	LMP, USS	13.0	NR
Mekuriyaw et al., 2020	Ethiopia	Case control	General population of pregnant women	Hospital	405	LMP, USS	NR	NR
Mochache et al., 2018	Kenya	Cohort	General population of pregnant women	NR	255	LMP, USS, SFH	11.0	NR

Mokuolu et al., 2002	Nigeria	Case control	General population of pregnant women	Hospital	171	LMP, USS, Ballard score	NR	NR
Mombo-ngoma et al., 2016	Benin Gabon Mozambique Tanzania	Cohort	General population of pregnant women	Hospital	4100	Ballard score	4.0	NR
Moodley et al., 2017	South Africa	Cohort	General population of pregnant women	NR	615	LMP, USS	18.0	NR
Muhayimana et al., 2017	Rwanda	Cohort	Preterm babies admitted	Hospital	125	NR	NR	-All the participants were preterm babies admitted neonatal intensive care unit<br>-Mortality rate was high (75%, 25%, and 2% for severe, moderate, and late preterm babies, respectively).<br>-The main morbidities were neonatal sepsis (88·8%), and RDS (62·4%), neonatal jaundice (21.6%) and asphyxia (17.6%)<br>-Morbidities and mortality was high as gestational age and birth weight decreased.
Muhe et al., 2019	Ethiopia	Cross-sectional	Preterm babies admitted	Hospital	4919	LMP, USS, Ballard score	NR	-78.3% of preterm babies were admitted.<br>-28.8% of admitted babies died compared to 0.7% in those not admitted.<br>-22·7% of preterm babies died, 89% of these deaths were for extremely preterm babies<br>-Causes of death were RDS (45%), neonatal infection (30%), combined sepsis, pneumonia, meningitis and birth asphyxia (14%).<br>-Hypothermia was the most common contributory cause of preterm mortality (69%)
Muhihi et al., 2016	Tanzania	Cohort	General population of pregnant women	Hospital and home delivery	19,269	LMP	16.0	NR

Muhumed et al., 2021	Ethiopia	Cross-sectional	General population of pregnant women	Hospital	600	LMP, USS	12.0	NR
Muti et al, 2015	Zimbabwe	Cross-sectional	General population of pregnant women	Hospital	289	NR	9.0	NR
Muwazi et al., 2014	Uganda	Cross-sectional	General population of pregnant women	Hospital	400	SFH	13.0	NR
Naidoo et al., 2016	South Africa	Cohort	General population of pregnant women	Hospital	250	LMP, USS, SFH	9.0	NR
Namazzi et al., 2020	Uganda	Cohort	Preterm babies admitted	Hospital	454	Ballard score	NR	-High incidence of neurodevelopmental disorders among preterm infants (20.4%) compared to term infants (7.5%). -High prevalence of malnutrition among preterm infants (18.6%) compared to term infants (2.1%).
N'Dao et al., 2006	Senegal	Cross-sectional	General population of pregnant women	NR	8273	LMP	18.0	NR
Ngandu et al., 2021	DRC South Africa	Cross-sectional	General population of pregnant women	Hospital	1084	NR	17.1 in DRC, 11.5 in South Africa	NR
Njim et al., 2016	Cameroon	Cross-sectional	General population of pregnant women	Hospital	4941	NR	10.0	NR
Njom Nlend et al., 2016	Cameroon	Cross-sectional	HIV positive pregnant women	NR	762	LMP, SFH, USS	9.0	NR
Nkyekyer et al., 2002	Ghana	Cohort	General population of pregnant women		1869	LMP, USS	14.0	NR
Nkyekyer et al., 2006	Ghana	Cohort	General population of pregnant women	Hospital	4731	LMP, USS, Ballard score	9.0	NR
Noble et al., 2005	Mozambique	Cross-sectional	General population of pregnant women	Hospital	497	NR	11.0	NR
Ojule et al., 2011	Nigeria	Case control	Primigravidae pregnant women	Hospital	1589	NR	5.0	NR

Okwako et al., 2017	Kenya	Cohort	Preterm babies admitted	Health facilities and home	175	NR	NR	-Mortality rate among preterm infants was 39.4%.<br>-Mortality rates were 70.4% for infants born <28 weeks, 50% for those born between 28 and 31 weeks and 24.5% for those born above 32 weeks.<br>-Predictors of mortality were GA>32 weeks (aHR0·4, 0·2-0·8), Emergency CS (aHR4·3, 1·9-9·7), Birthweight > 1000 (aHR0·3, 0·1-0·6), ANC attendance (aHR0·5, 0·3-0·9)
Oluwafemi et al., 2016	Nigeria	Cross-sectional	General population of pregnant women	Hospital	10,432	LMP, Ballard Score	15.4	-7.2% of preterm babies were admitted.<br>-Preterm case fatality rate was 5·4%.<br>-Extremely and very low birth weight babies were 250 times and 47.6 times more likely to die compared to term babies respectively.
Oluwole et al., 2020	Nigeria	Cross-sectional	General population of pregnant women	Hospital	206	LMP, USS	NR	NR
Olusanya et al., 2010	Nigeria	Cross-sectional	General population of pregnant women	Hospital	4314	LMP	20.0	NR
Omokhodion et al., 2010	Nigeria	Cross-sectional	General population of pregnant women	Hospital	1504	LMP	10.0	NR
Onwughara et al., 2020	South Africa	Cross-sectional	General population of pregnant women	Hospital	1758	LMP, USS	17.0	NR
Osman et al., 2001	Mozambique	Cohort	General population of pregnant women	Hospital	908	USS	15.0	NR
Osungbade et al., 2011	Nigeria	Cross-sectional	General population of pregnant women	Hospital	802	NR	16.0	NR
Padonou et al., 2014	Benin	Cross-sectional	General population of pregnant women	Health centres	526	Ballard Score	10.0	NR

Parek et al., 2011	Botswana	Cohort	General population of pregnant women	Hospital	16,219	LMP, SFH	20.0	NR
Patel et al., 2017	Kenya	Cohort	Preterm babies admitted	Hospital	88	NR	NR	-All participants were hospitalized preterm babies.<br>-11.4% of preterm babies admitted died.<br>-The mean duration of hospital stay was 37days<br>-The major causes of death were neonatal sepsis (50%), pulmonary hemorrhage (40%), pneumonia (10%)
Rakoto-Alson et al., 2010	Madagascar	Cohort	General population of pregnant women	NR	204	LMP, USS	21.0	NR
Ramdin et al., 2020	South Africa	Cohort	Preterm infants		56	LMP, Ballard Score	NR	-7.1% of preterm infants had developmental disability compared to none in term infants<br>-Developmental outcome, as assessed by the Bayley scales of infant and toddler development, was not different between late preterm infants and comparison peers.
Rayco-Solon et al., 2005	Gambia	Cohort	General population of pregnant women	NR	1916	Dubowitz	12.0	NR
Regasa et al, 2021	Ethiopia	Case control	General population of pregnant women	Hospital	358	NR	NR	NR
Rugumisa et al., 2020	Tanzania	Case control	General population of pregnant women	Hospital	222	LMP	17.0	NR
Saaka et al., 2013	Ghana	Cross-sectional	General population of pregnant women		524	USS	7.0	NR
Sania et al., 2017	South Africa	Cohort	HIV positive pregnant women	NR	571	LMP, USS	14.0	NR
Sania et al., 2015	Tanzania	RCT	HIV negative pregnant women	NR	6664	LMP	15.0	-Preterm babies were at a higher risk of neonatal mortality (RR=2.6) compared to term babies (RR=2.3).

Shava et al., 2019	Botswana	Cross-sectional	General population of pregnant women	Hospital	66,812	LMP, USS	19.0	NR
Siakwa et al., 2020	Ghana	Case control	General population of pregnant women	Hospital	1006	NR	NR	NR
Sigalla et al., 2017	Tanzania	Cohort	General population of pregnant women	Health facility & home	1112	USS	8.0	NR
Soroye et al., 2015	Tanzania	Cross-sectional	General population of pregnant women	Hospital	453	LMP	13.0	NR
Sow et al., 2018	Senegal	Cross-sectional	General population of pregnant women	Hospital	501	NR	28.0	NR
Stewart et al., 2019	Malawi	Cohort	General population of pregnant women	NR	1006	USS	7.0	NR
Stringer et al., 2010	Malawi Tanzania Zambia	RCT	HIV Positive pregnant women	NR	2428	SFH	25.0	NR
Suleiman et al., 2010	Nigeria	Cohort	Preterm babies admitted	Hospital	185	Ballard score, LMP	NR	-Participants were hospitalized preterm babies.<br>-12·2% of the hospitalized babies died
Sullivan et al., 1999	Malawi	Cohort	Primigravid pregnant women	Hospital	178	Dubowitz score	19.0	NR
Tadese et al., 2020	Ethiopia	Case control	General population of pregnant women	Hospital	414	NR	NR	NR
Tamambang et al., 2018	Cameroon	Cross-sectional	General population of pregnant women	Hospital	8056	NR	8.0	NR
Tamene et al., 2020	Ethiopia	Cross-sectional	Preterm babies admitted	Hospital	686	NR	NR	-Participants were hospitalized preterm babies.<br>-36·1% of these babies died<br>-None of the extremely preterm babies survived.
Teklay et al., 2018	Ethiopia	Case control	General population of pregnant women	Hospital	264	NR	NR	NR

Tembo et al., 2020	Zambia	Cohort	General population of pregnant women	Home and hospital	11,501	NR	5.0	NR
Ticconi et al., 2003	Zimbabwe	Cohort	General population of pregnant women	Hospital	986	LMP	29.0	NR
Turton et al., 2017	South Africa	Cohort	General population of pregnant women	NR	443	NR	32.0	NR
Ugochukwu et al., 2002	Nigeria	Cohort	Babies admitted	NR	699	Dubowitz	NR	NR
Umeora et al., 2004	Nigeria	Case control	General population of pregnant women	Hospital	261	NR	6.0	NR
Uwambaye et al., 2021	Rwanda	Case control	General population of pregnant women	NR	555	NR	NR	NR
Van den Broek et al, 2005	Malawi	Cohort	General population of pregnant women	53.2% hospital or health centre, 39.2% home and 7.5%- TBA hut	453	USS	20.0	NR
Van Den Broek et al., 2014	Malawi	Cohort	General population of pregnant women	Health centre, hospital, home	2149	USS	16.0	NR
Van der Merwe et al., 2011	South Africa	Cohort	HIV Positive pregnant women	NR	1093	LMP, USS, SFH	13.0	NR
Van zyl et al., 2018	South Africa	Cohort	Pregnant women with diabetes	Hospital	725	USS	33.0	NR
Wagura et al., 2018	Kenya	Cross-sectional	General population of pregnant women	Hospital	322	Finnstrom score	18.0	NR
Wakeyo et al., 2020	Ethiopia	Case control	General population of pregnant women	Hospital	244	LMP, USS	NR	NR
Watson-Jones et al., 2007	Tanzania	Cohort	General population of pregnant women	Hospital	1688	USS	12.0	NR
Woday et al., 2019	Ethiopia	Case control	General population of pregnant women	Hospital	417	LMP, USS, Ballard score	NR	NR
Yismaw et al., 2018	Ethiopia	Cross-sectional	Preterm babies admitted	Home (4.5%), Health center	516	NR	NR	-Participants were hospitalized preterm babies.

				(23.3), Hospital (72.3)				-28·8% of these babies died.
Young et al., 2012	Uganda	RCT	HIV Positive pregnant women	Hospital, Outside hospital	232	LMP, USS	18.0	NR
Zar et al., 2020	South Africa	Cohort	General population of pregnant women	NR	1137	LMP, USS, SFH	17.0	NR
Zash et al., 2018	Botswana	Cohort	HIV Positive pregnant women	Government maternity sites	3384	LMP	20.0	NR
Zewde et al., 2020	Ethiopia	Cross-sectional	General population of pregnant women	Hospital	325	LMP, USS	25.0	NR
Zini et al., 2019	Nigeria	Cohort	General population of pregnant women	Hospital	2200	LMP, USS	16.0	NR
LMP: Last Menstrual Period; NR: Not Reported; RCT: Randomized Controlled Trial; SFH: Symphysial fundal Height; USS: Ultrasound Sonography								
